# Humoral immune responses toward tumor-derived antigens in previously untreated patients with chronic lymphocytic leukemia

**DOI:** 10.18632/oncotarget.13712

**Published:** 2016-11-30

**Authors:** Valentina Griggio, Giorgia Mandili, Candida Vitale, Michela Capello, Paolo Macor, Sara Serra, Barbara Castella, Silvia Peola, Myriam Foglietta, Daniela Drandi, Paolaé Omed, Daniele Sblattero, Paola Cappello, Roberto Chiarle, Silvia Deaglio, Mario Boccadoro, Francesco Novelli, Massimo Massaia, Marta Coscia

**Affiliations:** ^1^ Division of Hematology, University of Torino, AOU Città della Salute e della Scienza di Torino, Torino, Italy; ^2^ Department of Molecular Biotechnology and Health Sciences, University of Torino, Torino, Italy; ^3^ Center for Experimental Research and Medical Studies (CeRMS), AOU Città della Salute e della Scienza di Torino, Torino, Italy; ^4^ Department of Life Sciences – University of Trieste, Trieste, Italy; ^5^ Department of Medical Sciences, University of Torino and Immunogenetics Unit - Human Genetics Foundation (HuGeF), Torino, Italy; ^6^ Molecular Biotechnology Center, Torino, Italy; ^7^ Department of Pathology, Boston Children's Hospital, Boston, Massachusetts, USA; ^8^ Service of Immunogenetics and Transplantation, AOU Città della Salute e della Scienza di Torino, Torino, Italy

**Keywords:** chronic lymphocytic leukemia, humoral responses, tumor antigens, serological proteomics, alpha-enolase

## Abstract

In chronic lymphocytic leukemia (CLL) the occurrence and the impact of antibody responses toward tumor-derived antigens are largely unexplored. Our serological proteomic data show that antibodies toward 47 identified antigens are detectable in 29 out of 35 patients (83%) with untreated CLL. The glycolytic enzyme alpha-enolase (ENO1) is the most frequently recognized antigen (i.e. 54% of CLL sera). We show that ENO1 is upregulated in the proliferating B-cell fraction of CLL lymph nodes. In CLL cells of the peripheral blood, ENO1 is exclusively expressed at the intracellular level, whereas it is exposed on the surface of apoptotic leukemic cells.

From the clinical standpoint, patients with progressive CLL show a higher number of antigen recognitions compared to patients with stable disease. Consistently, the anti-ENO1 antibodies are prevalent in sera from patients with progressive disease and their presence is predictive of a shorter time to first treatment. This clinical inefficacy associates with the inability of patients’ sera to trigger complement-dependent cytotoxicity and antibody-dependent cellular cytotoxicity against leukemic cells.

Together, these results indicate that antibody responses toward tumor-derived antigens are frequently detectable in sera from patients with CLL, but they are expression of a disrupted immune system and unable to hamper disease progression.

## INTRODUCTION

Immune dysfunctions are a key feature of chronic lymphocytic leukemia (CLL) and frequently result in clinically manifest complications contributing to patients’ morbidity and mortality, such as opportunistic infections and autoimmune conditions. The impairment of the host immune system also correlates with disease progression and may reflect the attempt of the leukemic cells to evade immune control [1].

Perturbations of both cellular and humoral immunity are observed in CLL patients. The T-cell compartment is highly abnormal, with changes in the phenotype and absolute number of circulating CD4+ and CD8+ T cells and with the accumulation of terminally differentiated effector memory T cells [2]. T lymphocytes express markers of exhaustion, such as upregulated PD-1, leading to a pronounced Th2 skewing of the T-cell responses [3], have reduced cytotoxic functions and an impaired formation of immune synapses with antigen (Ag) presenting cells [4]. Regulatory T cells are increased in the peripheral blood (PB) of patients with advanced disease [5], and Vγ9Vδ2 T cells display features of exhaustion and are negative prognosticator of a shorter time to first treatment (TTFT) [6].

Quantitative and qualitative changes of the humoral responses are also very common in patients with CLL. Hypogammaglobulinemia affects up to 85% of patients and its severity correlates with stage and duration of the disease and with the susceptibility to recurrent infections [1, 7]. On the other hand, haematopoietic-specific auto-antibodies (Ab) are frequently observed and autoimmune haemolitic anemia (AIHA) and/or immune thrombocytopenia are rather frequent complications. Autoimmunity is usually due to high affinity polyclonal immunoglobulin (Ig) G auto-Ab that are produced by the non-malignant B lymphocytes and recognize red cells- or platelets-derived auto-Ag [4, 8].

So far, little is known on the occurrence of humoral immune responses toward tumor-derived Ag in CLL. In a previous report, Krackhardt et al. detected circulating Ab recognizing 14 Ag derived from primary tumor samples in sera from patients with CLL [9]. However, the biologic functions and the clinical impact of spontaneously occurring humoral responses directed toward tumor-derived Ag have not been investigated.

Serological Proteome Analysis (SERPA) is a valuable method to detect Ag-specific Ab responses in human malignancies. SERPA combines electrophoretic separation of proteins from tumor cells, western blotting and mass spectrometric (MS) identification of Ag recognized by sera. SERPA allowed the identification of tumor-derived Ag able to trigger humoral immune responses in several solid [10–16] and hematologic tumors [17–20]. Dubovsky et al. have recently applied a modified SERPA to CLL to define new membrane-associated targets, and identified lymphocyte cytosolic protein 1 as an important factor in chemokine-induced migration of leukemic cells [21].

The aim of our study was to investigate the occurrence of humoral responses toward Ag derived from primary tumor cells in sera from previously untreated patients with CLL, also evaluating their cytotoxic function and the correlation with parameters of disease evolution.

## RESULTS

### Immunoreactivity of CLL sera toward CLL-derived Ag and protein identification

**Figure 1:**
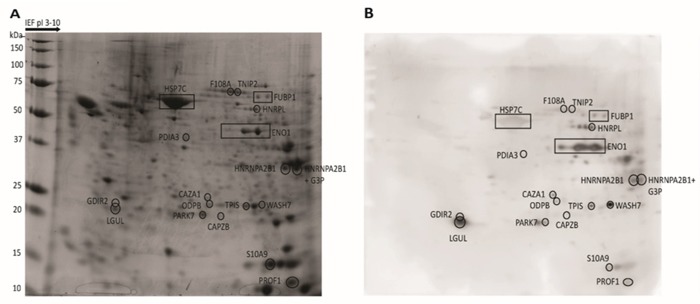
Two-dimensional map of proteins from CLL cells and corresponding immunoblotting map Total proteins extracted from CLL cells were separated by 2-DE, stained with Coomassie Blue **(A)**, or transferred on nitrocellulose membranes for WB and probed with CLL patient's autologous serum **(B)**. The proteins that could be assigned to Ag spots in the WB were excised from the gel and treated with trypsin. The resulting fragments were analyzed by MS. The location of proteins recognized by a representative CLL serum (patient 29) are indicated by boxes in the proteomic map and in the corresponding WB membrane.

Sera obtained from 35 patients with previously untreated CLL were individually screened for the presence of IgG-based immunoreactivity against proteins derived from autologous CLL cells (Figure 1A and 1B). To verify the CLL-specificity of the detection, 8 CLL proteomic maps (patients 05, 06, 10, 11, 12, 13, 16, 29) were individually probed with a different healthy donor's (HD) serum (data not shown). Overall, the 35 CLL sera recognized 144 Ag (mean:4.1, min:0, max:20), whereas the 8 HD sera recognized only 3 Ag (mean:0.4, min:0, max:1) (p=0.007).

The MS analyses of the 144 Ag spots produced by CLL sera led to the identification of 47 proteins that were recurrently recognized by patients’ sera. Table 1 summarizes the chemical features, spectrometric data, and frequency of recognition of 18 Ag, which were recognized by at least 3 patients’ sera. Additional proteins recognized by less than 3 patients’ sera are reported in Supplementary Table S1. Only 3 proteins (fructose-bisphosphate aldolase A [ALDOA], 60 kDa heat shock protein [HSP60] and vimentin [VIME]) were also recognized by 1 out of 8 (13%) HD sera. Sera from 29 out of 35 CLL patients (83%) exhibited IgG-based immunoreactivity toward at least 1 Ag (Table 2). Among these, 20 (69%) were polyreactive and recognized a median number of 5 Ag, with a peak of 20 Ag recognized by the serum of patient 22.

**Table 1:** Proteins recognized by at least 3 CLL sera

MW (kDa)/p*I*	Swiss-Prot Accession number	Protein name	Sequence coverage (%)	Metched peptides	Mascot score	Recurrence of antibodies (%)		
						CLL patients	Healthy Donors	p
47,481/7.10	Q6GMP2	α-Enolase (ENO1)	53	20	250	19 (54)	0	0.006
33,073/5.45	P52907	F-actin-capping protein subunit alpha-1 (CAZA1)	32	6	73	9 (26)	0	ns
36,201/8.57	P04406	Glyceraldehyde-3-phosphate dehydrogenase (G3P)	33	10	125	9 (26)	0	ns
39,550/6.20	P11177	Pyruvate dehydrogenase E1 component subunit beta, mitochondrial (ODPB)	43	11	93	8 (23)	0	ns
23,031/5.10	P52566	Rho GDP-dissociation inhibitor 2 (GDIR2)	56	9	93	7 (20)	0	ns
37,462/8.97	P22626	Heterogeneous nuclear ribonucleoproteins A2/B1 (HNRNPA2B1)	39	13	151	6 (17)	0	ns
53,676/5.06	P08670	Vimentin (VIME)	53	20	250	6 (17)	1 (13)	ns
42,052/5.29 42,108/5.31	Q96HG5 P63261	Actin, cytoplasmic 1 (ACTB) - Actin, cytoplasmic 2 (ACTG)	39	13	145	5 (14)	0	ns
31,616/5.36	P47756	F-actin-capping protein subunit beta (CAPZB)	36	8	98	5 (14)	0	ns
45,753/6.86	P14902	Indoleamine 2,3-dioxygenase 1 (I23O1)	23	8	66	5 (14)	0	ns
29,843/5.57	P35232	Prohibitin (PHB)	51	10	148	5 (14)	0	ns
61,187/5,70	P10809	60 kDa heat shock protein (HSP60)	37	14	154	4 (11)	1 (13)	ns
39,851/8.30	P04075	Fructose-bisphosphate aldolase A (ALDOA)	44	13	106	4 (11)	1 (13)	ns
23,569/5.43	P09211	Glutathione S-transferase P (GSTP1)	55	7	96	4 (11)	0	ns
71,082/8.52	P11142	Heat shock cognate 71 kDa protein (HSP7C)	37	16	197	3 (9)	0	ns
49,240/6.03	Q8NFZ5	TNFAIP3-interacting protein 2 (TNIP2)	17	8	69	3 (9)	0	ns
51,230/5.39	P61978	Heterogeneous nuclear ribonucleoprotein K (HNRNPK)	34	16	109	3 (9)	0	ns
64,720/8.46	P14866	Heterogeneous nuclear ribonucleoprotein L (HNRPL)	16	8	78	3 (9)	0	ns

MW, Molecular weight; p*I*, isoelectrical point. Sequence coverage (%) is the percentage of the protein primary sequence covered by peptides obtained by tripsyn digestion of the Ag spot. Matched peptides is the rate between the number of peptides corresponding to the primary sequence of the protein and the total number of peptides analyzed. Mascot score is a measure of the correspondence of peptides to the protein primary sequence.

**Table 2:** Immunoreactivity of patients with CLL

Patient	No of recognized proteins	Anti-ENO1 Ab	Binet	Disease state	IGHV	V-D-J	Stereotyped receptor subset	Cytogenetics by FISH	Hypogamma-globulinemia
01	2	+	A	stable	UM	1-18/6-19/4	#1	del(17p)	yes
02	1	-	A	stable	UM	1-18/3-3/6		del(13q)	
03	0	-			UM	3-23/4-23/4			
04	1	-	A	stable	UM	1-2/1-26/6	#28	del(11q)	yes
05	5	+	B	progression	M	4-34/3-3/4	#4	normal	yes
06	6	-		stable	M	5-51/2-2/5		del(13q)	
07	1	-	A	stable	n.d.			normal	yes
08	8	-	A	stable	M	3-33/3-22/4		del(17p)	yes
09	1	+	A	stable	M	4-34/5-5/6	#4	tris(12)	no
10	6	+	B	progression	M	3-64/6-6/4		del(13q)	yes
11	5	+	C	progression	UM	3-23/5-5/4		del(13q)	
12	4	-	A	stable	M	4-39/6-6/4		tris(12)	no
13	2	+	A	stable	M	5-51/2-2/6		del(11q)	yes
14	0	-			M	4-34/n.d./6		normal	no
15	3	+	B	stable	M	2-5/6-13/5		tris(12)	yes
16	3	+	A	progression	UM	4-59/3-22/6		normal	yes
17	0	-	A	stable	UM	3-33/3-3/6		normal	no
18	1	-	A	progression	UM			normal	no
19	0	-	A	stable	UM	5-a/6-19/4	#1	normal	no
20	0	-	B	stable	n.d.			del(17p)	yes
21	1	+	A	progression	M	2-70/5-24/4		tris(12)	yes
22	20	+	B	progression	UM	3-30/2-2/5		del(17p)	no
23	5	+	B	progression	UM	3-11/3-22/4		del(11q)	yes
24	2	+	B	stable	M	3-66/3-16/4		del(11q)	yes
25	12	+	B	progression	M	3-23/3-22/3		normal	no
26	10	+	A	stable	M	3-7/2-2/4		normal	yes
27	1	+	A	stable	UM	4-59/3-22/6		normal	
28	0	-	B	stable	UM	3-33/4-17/4		del(13q)	no
29	19	+	B	progression	M	3-74/3-10/4		normal	no
30	1	-	A	stable	M	3-53/6-13/4		del(13q)	no
31	1	+			UM	4-39/5-12/5		normal	
32	5	+	A	stable	UM	1-69/3-3/6	#7	normal	no
33	9	+	A	stable	M	7-4/7-27/4		del(13q)	yes
34	6	-	A	stable	UM	3-53/n.d./4		del(13q)	yes
35	3	-	A	stable	UM	3-9/6-19/6		tris(12)	no

### Sera from progressive CLL are more immunoreactive than sera from stable CLL

**Figure 2:**
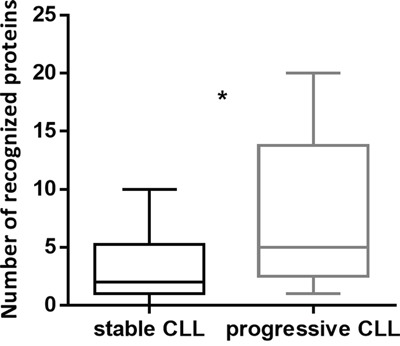
The number of recognized Ag was significantly higher in patients with progressive CLL than in patients with stable disease The number of recognized Ag was correlated with parameters of disease progression collected at the time of SERPA. Disease progression was evaluated according to IWCLL/NCI-WG 2008 guidelines for CLL. Patients with progressive CLL (n=10) recognized a significantly higher number of spots compared to patients with stable CLL (n=22) (p=0.01). Box and whiskers plots represent median values, first and third quartiles, and minimum and maximum values for each dataset.

The immunoreactive status of patients was not associated with gender, age, Rai or Binet stage, lymphocytosis, monocyte and platelet counts, hemoglobin, β2-microglobulin or lactate dehydrogenase values, percentage of CD38+ or ZAP70+ cells, immunoglobulin heavy chain variable regions (IGHV)-mutational status, fluorescence *in situ* hybridization data, hypogammaglobulinemia, autoimmunity, concurrent infections or allergies. Our cohort included 10 patients in active disease progression and 22 patients with a stable disease. For the remaining 3 patients, the disease status at the time of SERPA was not available. Interestingly, we found that patients with progressive CLL showed a significantly higher number of Ag recognitions compared to patients with stable disease (p=0.01) (Figure 2). Overall, median TTFT of patients was 29 months and median overall survival (OS) was not reached at the median follow-up of 81 months. The immunoreactivity status was not a statistically significant TTFT or OS predictor.

### Circulating Ab are produced by the polyclonal B-cell population and not by the leukemic clone

To determine whether the circulating Ab detected by SERPA in patients’ sera were partly produced by a class-switched sub-clone derived from the leukemic clone, we first analyzed the IGHV repertoire of patients who shared the same Ag recognitions. Patients with similar immunoreactivities did not exhibit the same IGHV rearrangements. In addition, the analysis of the complementarity determining region 3 (CDR3) revealed that 6 out of 35 CLL patients displayed stereotyped B-cell receptor (BCR) and belonged to 4 subsets, with no association between stereotypy and Ag recognition. As a confirmatory evidence, we cloned and produced a soluble derivative of the leukemic BCR (ScFv-Fc) from patient 10 and blotted it in parallel with the whole patient's serum on two identical autologous proteomic maps. The patient's serum recognized 5 Ag, but none of these was also recognized by the autologous tumor-derived ScFv-Fc (Supplementary Figure S1). Taken together, these results indicate that the Ab detected by SERPA are entirely produced by the normal B-cell population, and do not include a soluble fraction of the leukemic BCR.

### Alpha-enolase (ENO1) is the most frequently recognized Ag and is overexpressed by proliferating CLL cells of the LN

ENO1 was the most relevant Ag recognized by patients’ sera, since ENO1-specific circulating Ab were detectable in 19 out of 35 (54%) CLL sera and in none of the HD sera (p=0.006) (Table 1). To confirm the MS identification of the protein, we incubated the proteomic maps obtained from the tumor cells of 10 anti-ENO1 Ab+ (patients 22, 23, 24, 25, 26, 27, 29, 31, 32, 33) and 4 anti-ENO1 Ab- patients (patients 28, 30, 34, 35) with a commercially available anti-ENO1 polyclonal Ab. The anti-ENO1 Ab generated the same Ag spots produced by the sera of patients with CLL (Supplementary Figure S2).

**Figure 3:**
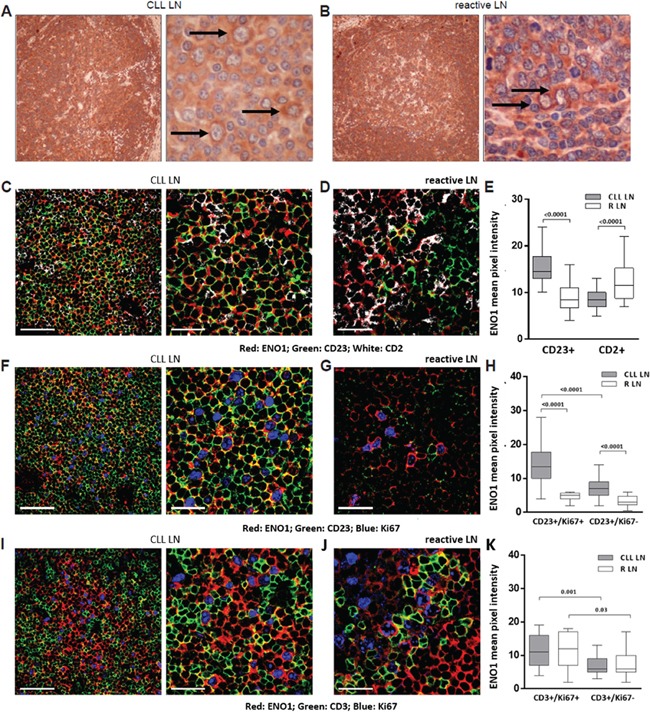
In lymph node ENO1 is significantly more expressed by proliferating CLL cells ENO1 expression was evaluated by immunohistochemistry and immunofluorescence confocal microscopy on CLL and R LN sections. Anti-ENO1 immunostaining of a representative CLL LN and R LN section was performed using an anti-mouse HRP-conjugated secondary Ab and 3,3′-diaminobenzidine (brown signal). Black arrows indicate paraimmunoblasts **(A)** and centroblasts **(B)**. Original magnification x4 (left panels) and x40 (right panels). Images were obtained using a Olympus BX41 microscope, equipped with Nikon Coolpix camera. Triple staining of representative CLL and R LN sections with anti-ENO1 (red), anti-CD23 (green) and anti-CD2 (white). Original magnification x63; zoom factor of 2.5 for images on the right. Scale bars represent 50 μm and 25 μm, respectively **(C, D)**. Cumulative data of ENO1 mean pixel intensity (arbitrary units) confirmed a significantly higher intensity of ENO1 expression in close proximity of B cells in CLL LN and T cells in R LN (always p<0.0001) **(E)**. Triple staining of representative CLL and R LN sections with anti-ENO1 (red), anti-CD23 (green) and anti-Ki67 (blue). Original magnification x63; zoom factor of 2.5 for images on the right. Scale bars represent 50 μm and 25 μm, respectively **(F, G)**. Quantitative measurements of ENO1 mean pixel intensity (arbitrary units) confirmed that CD23+/Ki67+ proliferating cells of the CLL LN expressed significantly higher level of ENO1 compared to the CD23+/Ki67- resting fraction (p<0.0001) and to CD23+/Ki67+ proliferating B cells of the R LN (p<0.0001) **(H)**. Triple staining of representative CLL LN and R LN sections with anti-ENO1 (red), anti-CD3 (green) and anti-Ki67 (blue). Original magnification x63; zoom factor of 2.5 for images on the right. Scale bars represent 50 μm and 25 μm, respectively **(I, J)**. Cumulative analysis of ENO1 mean pixel intensity (arbitrary units) confirmed that the CD3+/Ki67+ proliferating fraction of the T-cell compartment expressed significantly higher levels of ENO1 than CD3+/Ki67- resting fraction, in both CLL and R LN (p<0.001 and 0.03 respectively) **(K)**. For cumulative data, at least 3 randomly chosen fields from 3 different samples were analyzed. Samples were analyzed using a TCS SP5 laser scanning confocal microscope (Leica Microsystems) with an oil immersion 63x/1.5 objective lens, images were acquired with LAS AF Version Lite 2.4 software and processed with Photoshop (Adobe Systems). ENO1 mean pixel intensity was calculated with ImageJ software ( http://rsbweb.nih.gov/ij/). Box and whiskers plots represent median values, first and third quartiles, and minimum and maximum values for each dataset. Statistical analysis was performed using Mann-Whitney *U* test.

ENO1 expression pattern was investigated by immunohistochemistry and multicolor immunofluorescence confocal microscopy in lymph node (LN) sections obtained from 3 CLL and 3 reactive (R) LN. The CLL LN displayed an almost complete effacement of the normal architecture by CLL cells and evidence of morphologically distinct pseudo-follicles, comprising areas rich in prolymphocytes and paraimmunoblasts. The anti-ENO1 Ab stained all CLL and R LN, and higher magnification indicated an increased expression in correspondence of proliferating cells of both CLL and R LN sections, at least on the basis of cell morphology (Figure 3A and 3B). Multicolor tissue immunofluorescence was then used to determine which cells were mostly ENO1+. The anti-ENO1 Ab was combined with anti-CD2 and anti-CD23 Ab to detect T and B cells, respectively. In the CLL LN ENO1 reactivity was mostly associated to the CD23+ population, while in the R LN it was mostly associated to the CD2+ T cells (Figure 3C–3E). Ki67 was then used to identify proliferating cells in both tissues. Within the CLL LN a significant proportion of proliferating B cells was apparent and these cells were intensely ENO1+. Conversely, in the reactive LN the most of the proliferating cells were CD2+, and they also showed ENO1 reactivity, suggesting that ENO1 is highly expressed by proliferating cells, independently of the lineage (Figure 3D–3K).

### ENO1 is transferred on the surface of CLL cells undergoing apoptosis

**Figure 4:**
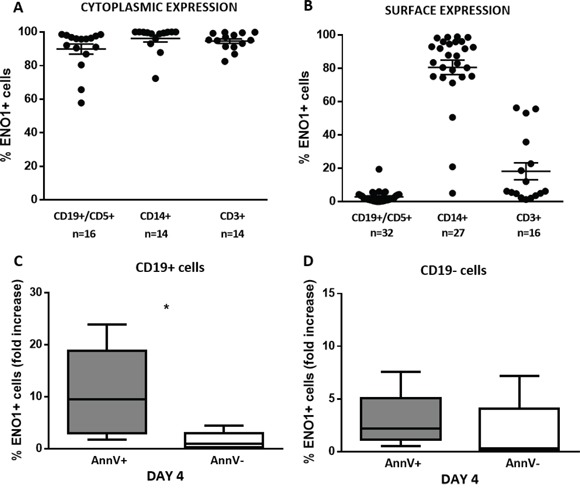
ENO1 is translocated on the surface of apoptotic CD19+ cells ENO1 expression was evaluated in PBMC isolated from patients with CLL, at baseline conditions and after 4-day *in vitro* culture by flow cytometry. **A**. ENO1 cytoplasmic expression. High percentages of ENO1 were expressed in the cytoplasm of CD19+/CD5+ CLL cells, CD14+ monocytes and CD3+ T cells. **B**. ENO1 surface expression. ENO1 was not expressed on the surface of CD19+/CD5+ CLL cells, whereas it was widely expressed by CD14+ monocytes and in a proportion of CD3+ lymphocytes. **C, D**. Surface ENO1 fold increase expression in CD19+ and CD19- cell fractions. Fold increase was calculated for each patient as a ratio between the percentage of CD19+/ENO1+ detected after 4-day *in vitro* culture and the percentage of CD19+/ENO1+ cells at baseline. After 4 days of culture, the fold increase in ENO1 expression was significantly higher in the apoptotic (AnnV+) than in the viable (AnnV-) fraction of CD19+ cells (p=0.02) (C). By contrast, there was no difference in the fold increase of ENO1 expression between the apoptotic (AnnV+) and the viable (AnnV-) fraction of CD19- cells (D). Box and whiskers plots represent median values, first and third quartiles, and minimum and maximum values for each dataset.

ENO1 expression was also evaluated in the PB compartment. First, we compared the intracellular expression of ENO1 in PB mononuclear cells (PBMC) subpopulations from CLL patients. Cytofluorimetric analysis showed that CD19+/CD5+ CLL cells, CD14+ monocytes, and CD3+ T lymphocytes expressed ENO1 at the intracellular level (Figure 4A).

We then evaluated the membrane expression of ENO1. ENO1 was not expressed on the surface of CD19+/CD5+ leukemic cells, whereas it was expressed to higher extent by CD14+ monocytes, and in a proportion of CD3+ T lymphocytes (Figure 4B). After 4 days of *in vitro* culture, the mean fold increase in the percentage of cells expressing ENO1 on the surface was significantly higher in the apoptotic (Annexin-V+ [AnnV+]) than in the viable (AnnV-) fraction of CD19+ cells (p=0.02) (Figure 4C). This difference in ENO1 surface expression (mean fold increase) was not observed between the apoptotic and viable fraction of CD19- cells (Figure 4D).

### Immunoreactivity toward ENO1 associates with CLL progression and shorter TTFT

**Figure 5:**
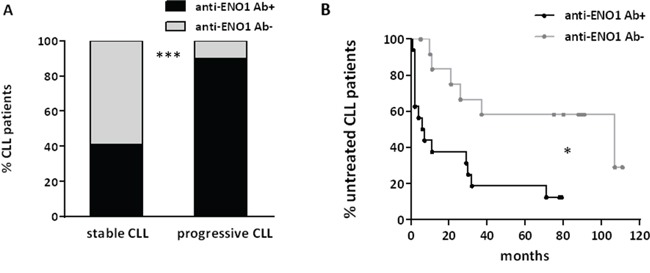
Anti-ENO1 Ab reactivity is associated with parameters of progressive disease and with a shorter TTFT in patients with CLL The presence of circulating anti-ENO1 Ab in sera of patients with CLL was correlated with parameters of disease progression and TTFT. Anti-ENO1 Ab were more frequently detected in sera from patients with progressive CLL than in sera from patients with stable CLL (p<0.0001) **(A)**. Kaplan-Meier survival curves for TTFT in our cohort of patients stratified on the basis of anti-ENO1 reactivity are shown. The presence of anti-ENO1 Ab correlated with shorter TTFT (ENO1 Ab+, n=19; ENO1 Ab-, n=16) (p=0.01) **(B)**.

The presence of circulating anti-ENO1 Ab was not associated with the occurrence of clinically evident autoimmune manifestations. By contrast, ENO1 reactivity was significantly associated to parameters of disease progression such as higher lymphocyte counts (p=0.02) and lower platelet numbers (p=0.007) (Supplementary Figure S3A and S3B). Similarly, anti-ENO1 Ab (Figure 5A) were more frequently detected in sera from patients with progressive disease than in sera from patients with stable disease (p<0.0001). As expected, log-rank test indicated that the presence of anti-ENO1 Ab in patients’ sera was a negative TTFT predictor in univariate analysis: the median TTFT of anti-ENO1 Ab+ CLL patients was 7 months, whereas that of anti-ENO1 Ab- CLL patients was 107 months (p=0.01) (Figure 5B). Lastly, we observed that the total level of serum IgG was significantly lower in anti-ENO1 Ab+ compared to anti-ENO1 Ab- CLL sera (p=0.02) (Supplementary Figure S4).

### Serum Ab are not able to trigger complement dependent cytotoxicity (CDC) and antibody dependent cellular cytotoxicity (ADCC)

**Figure 6:**
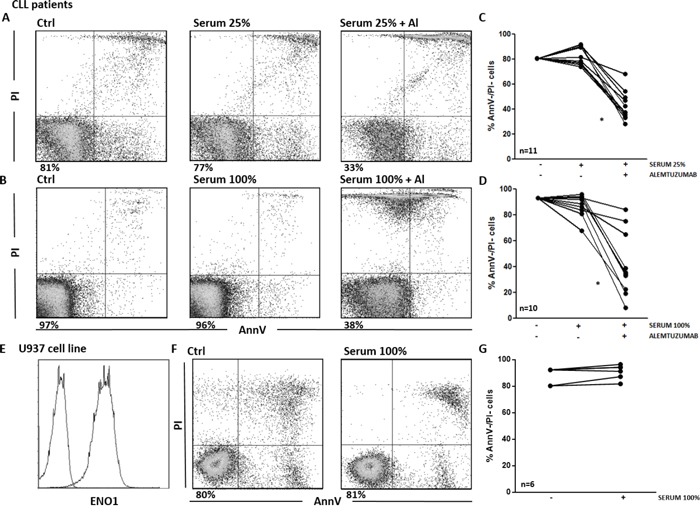
Serum Ab are not able to trigger CDC toward tumor cells **A-D**. Viability of CLL cells incubated for 1 hour with different concentrations of CLL serum, in the presence or in the absence of alemtuzumab. Cell viability was determined by Ann-V/PI staining and cytofluorimetric analysis on CLL cells purified from patients’ samples. **A, B**. Representative analyses of AnnV/PI expression on CLL cells left untreated or incubated with patient's serum at 25% (A) and 100% (B) concentration, in the presence or in the absence of alemtuzumab. Indicated is the percentage of AnnV/PI double negative (AnnV-/PI-) cells. **C, D**. Percentage of AnnV-/PI- viable CLL cells. Line plots show that cell viability was significantly reduced when CLL cells were exposed to alemtuzumab (*p*<0.01). By contrast, there was no significant decrease in cell viability when CLL cells were exposed to sera alone, both at the 25% (C) and 100% (D) concentrations. **E-G**. CDC assay on the U937 cell line. **E**. ENO1 surface expression. Representative analyses of ENO1 expression on U937 cells. **F**. Representative analyses of AnnV/PI expression on U937 cells left untreated or incubated with ENO1-Ab+ patients’ sera at 100% concentration. Indicated is the percentage of AnnV-/PI- cells. **G**. Percentage of viable AnnV-/PI- U937 cells. Line plot shows no significant decrease in cell viability after exposure of U937 cells to 6 ENO1-Ab+ sera from CLL patients.

Next, we asked whether the circulating Ab identified in patients sera were effective in triggering CDC. We first tested the efficacy of our samples of CLL sera as source of complement. As expected, the viability of CLL cells exposed to the monoclonal Ab alemtuzumab was significantly reduced compared to untreated controls (p<0.01). By contrast, we did not observe any significant decrease in cell viability when CLL cells were exposed to patients’ sera, in the absence of alemtuzumab (Figure 6A–6D). Flow cytometry revealed that there was no deposition of the complement component 4 on the surface of CLL cells after incubation with patients’ sera (data not shown). Overall, these data show that the circulating Ab detected by SERPA in CLL sera are not able to induce CDC toward CLL cells.

We then performed CDC assays with sera from anti-ENO1 Ab+ patients and the U937 cell line, which expresses high levels of surface ENO1 (Figure 6E). Again, no modification of cell viability was observed when U937 cells were treated with anti-ENO1 Ab+ sera, even when sera were used at 100% concentration (Figure 6F and 6G). These data demonstrate that the circulating Ab do not trigger CDC at the *in vivo* concentrations, even when the cognate Ag is highly expressed on the surface of the target cells.

**Figure 7:**
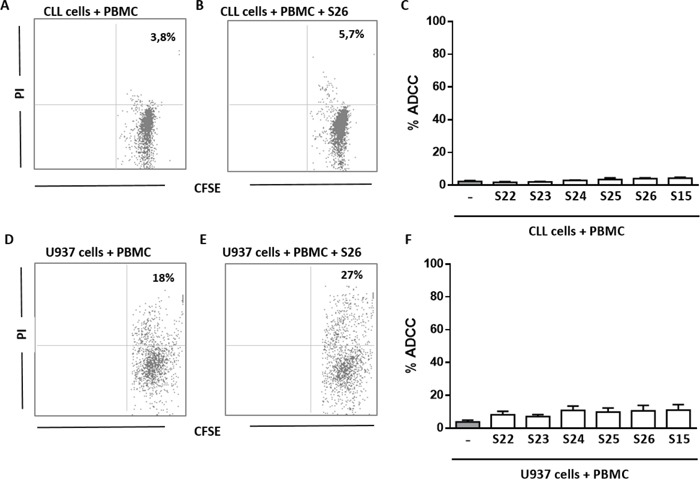
Anti-ENO1 Ab are not able to trigger ADCC against leukemic cells **A-C**. % of ADCC of CFSE labelled CLL cells co-cultured for 18 hours with HD PBMC, in presence or absence of 10% diluted anti-ENO1 Ab+ serum. Cell lysis was determined by PI staining and flow cytometry. % of ADCC was calculated by the formula % PI (T + E + serum) - % PI (T)/% PI (triton X treated T) – % PI (T). **A, B**. Representative analysis of CFSE/PI expression on CLL cells (gated on CFSE+ cells) purified from one patient's sample co-cultured with HD PBMC, used as effector cells, in the absence (A) or presence (B) of anti-ENO1 Ab+ serum at 10% concentration. Indicated is the % of CFSE/PI double positive cells. **C**. Bar graph show no significant differences in the % of ADCC of primary CLL cells cultured with PBMC and 10% diluted anti-ENO1 Ab+ serum (n=6) compared to target cells cultured with PBMC alone, in the absence of serum. **D-F**. ADCC assay on U937 cell line. **D, E**. Representative analysis of CFSE/PI expression on U937 cells (gated on CFSE+ cells) co-cultured with HD PBMC, used as effector cells, in the absence (D) or presence (E) of anti-ENO1 Ab+ serum at 10% concentration. Indicated is the % of CFSE/PI double positive cells. **F**. Bar graph show no significant differences in the % of ADCC of U937 cells cultured with PBMC and 10% diluted anti-ENO1 Ab+ serum (n=6) compared to target cells cultured with PBMC alone, in the absence of serum. Mean results from three different experiments.

In the last set of experiments, we tested the ability of anti-ENO1 Ab+ sera to trigger ADCC toward both the U937 cell line and primary CLL cells. We found that the % of ADCC of U937 cells cultured for 18 hours in the presence of 10% diluted anti-ENO1 Ab+ serum and HD PBMC, used as effector cells, was not significantly increased compared to target cells cultured with PBMC alone, in the absence of serum. Similar results were obtained using CLL cells as targets (Figure 7A–7D). Therefore, we can conclude that the circulating Ab detected by SERPA are unable to trigger ADCC against leukemic cells.

## DISCUSSION

In CLL, cellular immune responses are unable to respond to the tumor and they can even support disease evolution [22]. The impact of humoral responses toward tumor-derived Ag in CLL is largely unexplored. Through the use of SERPA we show that 83% of CLL sera have circulating Ab toward at least one CLL-derived Ag. Interestingly, we observe that sera from progressive CLL are significantly more immunoreactive than sera from stable CLL.

The circulating Ab detected by SERPA are expression of an immune response generated by the residual polyclonal B-cell population toward self-Ag. We do not observe shared Ag recognition between a representative CLL serum and the ScFv-Fc derived from the autologous leukemic BCR. On the other hand, patients who share the same Ag recognitions do not show recurrent IGHV rearrangements or BCR stereotypies. Autoimmune phenomena are rather frequent complications of CLL and mostly result in autoimmune cytopenias [23]. A clear association between autoimmune cytopenias and poor prognostic variables, such as high β2-microglobulin, CD38 and ZAP70 expression, and unmutated IGHV status, has already been described [24–26]. Moreover, direct antiglobulin test positivity, even in the absence of overt AIHA, associates with adverse prognostic factors and shorter OS [27]. In our cohort, we observe a significantly higher degree of immunoreactivity in patients with progressive CLL than in patients with stable disease, even though the immunoreactive status is not related to adverse prognostic factors.

MS analyses show that the Ab detected by SERPA mostly recognize intracellular Ag, such as glycolytic enzymes, cytoskeletal elements and ribonucleoproteins. Among others, ENO1, a glycolytic enzyme, is the most relevant Ag, detected in 19 out of 35 (54%) CLL sera. The overexpression of ENO1 is associated with tumor development through a process known as Warburg effect [28]. The Warburg effect is a metabolic reprogramming, which consists in a positive regulation exerted by the hypoxia-inducible factor (HIF) on the expression of glycolytic enzymes, such as ENO1, occurring when cancer cells are exposed to hypoxic conditions [29, 30]. Interestingly, CLL cells express the oxygen-regulated HIF-1α subunit even under normoxia [31], and the expression and the transcriptional activity of HIF-1α are further upregulated by exposure of CLL cells to stromal cells [32]. In the LN, proliferating CLL cells are confined to specialized structures called pseudo-follicles, where they interact with T lymphocytes and stromal cells. Our data show that ENO1 is more expressed in the leukemic cells of the CLL LN than in the normal B cells of the R LN, and confirm that it is more expressed by the proliferating B cells of the pseudo-follicles than by the resting B-cell fraction.

Due to its tumor-related overexpression and ability to induce humoral and/or cellular immune responses, ENO1 has already been classified as a tumor-associated Ag in solid cancers, such as pancreatic ductal adenocarcinoma (PDA) [12, 33]. Cappello et al. have reported that a DNA vaccine coding for ENO1 can elicit anti-ENO1 IgG Ab which are able to induce the killing of murine PDA cells by CDC [33]. Unexpectedly, in our cohort of CLL patients, the presence of anti-ENO1 Ab is prevalent in sera from patients with progressive CLL and is predictive of a shorter TTFT. One possible reason of the different clinical impact exerted by anti-ENO1 Ab in solid tumors and in CLL is that ENO1 is highly expressed at the intracellular level, but is not expressed on the cell membrane. It is not clear how intracellular self-Ag can become immunogenic and trigger humoral responses. One possible explanation is that overexpressed self-proteins are exposed during the apoptotic turnover of the leukemic cells, thus becoming visible and capable of inducing autoreactive immune responses. This hypothesis is corroborated by our data showing that after 4 days of *in vitro* culture, the mean fold increase in the percentage of cells expressing ENO1 on the surface is significantly higher in the apoptotic than in the viable fraction of CD19+ cells. The higher frequency of immunoreactivities detected by SERPA in the sera of patients with progressive CLL can be explained by the observation that in advanced-stage CLL the malignant clone, which in early-stage is arrested in G0/G1, evolve into an autonomously proliferating cell population showing a greater ability to enter spontaneous apoptosis [34]. In the appropriate context, such as in the spleen and LN, where T cells may induce the activation and Ag presentation by B cells, this turnover potentially leads to a greater exposition of intracellular overexpressed Ag and to the production of autoreactive Ab.

To functionally characterize the humoral responses detected by SERPA we evaluated the *in vitro* ability of patients’ sera to kill primary CLL cells by CDC or ADCC. Deficiencies or reduced levels in one or more complement components have already been reported in CLL patients [35, 36]. However, we demonstrated that CLL sera behave as proper source of complement, in inducing alemtuzumab-mediated CDC as effectively as HD sera. By contrast, in the absence of alemtuzumab, polyreactive CLL sera are not able to induce complement deposition on the surface of CLL cells and do not trigger CDC toward primary tumor cells. Similarly, CLL sera do not trigger ADCC against CLL cells, in the presence of PBMC isolated from HD used as effector cells. A further demonstration of the functional incompetence of circulating Ab detected by SERPA is the observation that CLL patients with immunoreacitive sera, even those with polyreactive sera, lack clinically manifest autoimmune phenomena. A number of reasons may underlie this functional incompetence. First of all, the tumor Ag recognized by the circulating Ab are mainly intracellular proteins and therefore are not optimal targets for an Ab-mediated immune response. However, we do not observe any cytotoxicity even when the U937 cell line, which expresses high levels of surface ENO1, is used as target for the CDC and ADCC assays. In line with these results is the observation that the absolute number of monocytes, which also express high levels of surface ENO1, is not decreased in the PB of CLL patients with anti-ENO1 circulating Ab (data not shown). These findings show that Ab-related issues, such as low sera concentrations of the circulating Ab and/or low binding affinity toward the corresponding Ag, may also contribute to the functional incompetence of the humoral responses detected by SERPA.

Overall, our results show that the Ab responses detected by SERPA are an epiphenomenon of a disrupted immune system, which is unable to control disease evolution. It has already been reported that the chronic inflammation state determined by an enhanced and persistent activation of humoral immune responses, in combination with the suppressed cellular anti-tumor immunity, may favour the tumor progression and support disease evolution [37]. In this context, it will be of interest to determine the immunomodulatory properties of the new BCR inhibitors, which have now been introduced in the clinical practice for the treatment of CLL. Current studies already show a recovery of humoral immunity and normal B-cell numbers in patients on ibrutinib, leading to a decrease in the rate of infections [38]. This observation, together with the promising results obtained in experimental models of autoimmune diseases [39, 40], support the hypothesis of an ibrutinib-induced immunomodulation which may contribute to tumor control.

## MATERIALS AND METHODS

### Patient population

PB samples were collected from a total of 86 patients with untreated CLL, after their informed consent, in accordance with the Declaration of Helsinki and approval by the local institutional review board. Sections of LN infiltrated by CLL cells and reactive R LN were obtained from the Department of Medical Sciences of the University of Torino, Italy. The diagnosis and progression of CLL were defined according to International Workshop on CLL-National Cancer Institute (IWCLL/NCI-WG) guidelines [41]. The disease was defined stable in absence of signs of progression. A cohort of 35 patients with CLL was analyzed by SERPA. Patients’ biological and clinical features were collected, when available, by chart review (Supplementary Table S2). The median follow-up of all patients was 81 months. The IGVH mutational status and the CDR3 clustering analyses were performed as previously reported [42, 43]. The control group consisted of 12 HD kindly provided by the local blood bank.

### Cells and serum samples

PBMC and purified B lymphocytes were prepared as described [32]. Serum samples were isolated from PB by centrifugation and stored at -80°C until use. In selected experiments, the human cell line U937 (ATCC, CRL 1593.2) was used.

### Sample preparation and 2-Dimension Electrophoresis (2-DE)

Pellets obtained from at least 10^7^ purified CLL cells were solubilized in lysis buffer (urea 9M, CHAPS 4%, Na_3_VO_4_ 1mM, DTT 80mM, protease inhibitors and nuclease). Samples were spun down at 13800 g for 10 minutes at 4°C. The clear supernatant was quantified with DC Protein assay kit (Bio-Rad, Hercules, CA, USA). 2-DE on ready-made IPG strip (7-cm IPG strips, pH 3-10NL; Bio-Rad) was performed essentially as described [44]. The 2-DE maps were obtained in duplicate and stained with Coomassie Blue or transferred onto a nitrocellulose membrane (GE Healthcare Biosciences GmbH, Uppsala, Sweden) for serological analysis. The 2-D gel images were acquired using the ChemiDoc MP system (Bio-Rad).

### Western blot analysis

The membrane was incubated overnight at 4°C with serum, as primary Ab at working dilution of 1:100, with a single chain variable fragment-fragment crystallisable region (ScFv-Fc) or with an anti-ENO1 Ab (Santa Cruz Biotechnology, Inc; CA, USA). Then the membrane was incubated with an anti-human (Santa Cruz Biotechnology, Inc) or anti-mouse (GE Healthcare Biosciences GmbH) IgG HRP-conjugated Abs. Images were acquired by the ChemiDoc MP system (Bio-Rad).

### Protein identification by MS

MS analysis was performed as described [44]. The 25 most intense masses were used for database searches against the Swiss-Prot database using the free search program Mascot ( http://www.matrixscience.com). Only proteins with a Mascot score greater than 56 were taken into consideration.

### ScFv-Fc production

VH and VL genes were amplified with specific primers from CLL derived cDNA as already described [45]. V genes were assembled as ScFv and cloned in fusion with the human Fc region (as scFv-Fc) in the pUCOE vector [46]. CHO-S cell line was transfected and stable clones was obtained for ScFv-Fc expression. Ab produced in cell culture supernatants were purified using a protein A/G column.

### Flow cytometry

ENO1 surface and intracytoplasmic expression was evaluated cytofluorimetrically on B cells, T cells and monocytes in PB samples as reported in Supplemental Methods.

### Cell culture and viability assay

PBMC from patients with CLL were cultured as described [32]. The percentage of viable and apoptotic cells was determined by Annexin-V (AnnV) or AnnV/Propidium Iodide (PI) staining with the MEBCYTO-Apoptosis Kit (MBL Medical and Biological Laboratories, Naka-ku Nagoya, Japan).

### Immunohistochemistry and confocal immunofluorescent microscopy

Cell morphology and numbers were studied by Giemsa staining. For immunocytochemistry, coverslips were stained as detailed in Supplemental Methods. Formalin-fixed, paraffin-embedded sections of CLL or R LN were stained and analyzed by light microscopy as already described [47] (details in Supplemental Methods).

### CDC and ADCC assays

For CDC, purified CLL cells (2 × 10^5^) were incubated in presence or absence of alemtuzumab (10 μg/ml) for 30 minutes at room temperature. CLL cells were then washed with PBS and incubated with selected patients’ sera at different dilutions for 60 minutes at 37°C. U937 cells were incubated only with CLL patients’ sera. Cell viability was evaluated using AnnV/PI staining (details in Supplemental Methods). For ADCC, purified CLL cells or U937 cells, used as target cells (T), were labeled with CFSE (5 μM) for 30 minutes at 37 °C. CLL cells and U937 cells were then incubated with PBMC from healthy donors, used as effector cells (E) for 18 hours at 30:1 E:T ratio, in the presence or absence of 10% diluted CLL patients’ sera. At the end of the co-culture target cells viability was evaluated by PI staining. % of ADCC was calculated as follow: % PI (T + E + serum) - % PI (T)/% PI (triton X treated T) – % PI (T).

### Statistical analysis

Statistical analyses were performed with GraphPad Prism (San Diego, CA, USA). Continuous variables were compared by Mann-Whitney *U* (unpaired data) or Wilcoxon signed rank (paired data) tests. The χ^2^ test was used in case of dichotomous variables. OS and TTFT were defined as the time between the date of SERPA and, respectively, the date of death or last follow-up and the date of first-line treatment or the last follow-up. OS and TTFT were estimated by the Kaplan-Meier method and the difference between groups was assessed by log-rank test. A p value <.05 was considered significant.

## SUPPLEMENTARY MATERIALS FIGURES AND TABLES




