# Supplementary material for: Humoral immune responses toward tumor-derived antigens in previously untreated patients with chronic lymphocytic leukemia
Source: Oncotarget. 2016 Nov 30;8(2):3274–88. doi: 10.18632/oncotarget.13712 (PMC5356881; doi:10.18632/oncotarget.13712)
Supplement: Supplementary file 1 [file oncotarget-08-3274-s001.pdf]

Humoral immune responses toward tumor-derived antigens in previously untreated patients with chronic lymphocytic leukemia

SUPPLEMENTARY DATA

SUPPLEMENTARY METHODS

Flow cytometry

Two- and three-color flow cytometry was performed with a FACSCalibur and CELLQuest software (Becton Dickinson, Mountain View, CA, USA). Data were analyzed with FlowJo software (Tree Star, Inc, Ashland, OR, USA). CLL cells were identified and characterized with the use of anti-CD5-APC (DAKO SpA, Milano, Italy), anti-CD19-PerCP (Beckman Coulter, Milano, Italy), anti-CD19-Vio 770 (Miltenyi Biotec, Bologna, Italy) or anti-CD19-PE (Beckman Coulter, Milano, Italy) monoclonal Ab. To determine the percentage of ENO1 positive cells, PBMC from CLL patients were washed in PBS and stained with anti-CD19-Vio770 (Miltenyi Biotec, Bologna, Italy), anti-CD5-APC (Miltenyi Biotec), anti-CD3-PerCP (Dako Spa), or anti-CD14-PE (BD Biosciences, San José, CA, USA) monoclonal Ab in association with an anti-ENO1 primary Ab (H-300, rabbit polyclonal IgG, Santa Cruz Biotechnology, Inc; CA, USA) and a secondary donkey anti-rabbit IgG FITC conjugated Ab (BioLegend, San Diego, CA, USA) or a goat anti-rabbit IgG APC conjugated (LifeTechnologies, Monza, Italy) Ab. For intracytoplasmic ENO1 detection was used the Fix and Perm Cell Fixation and Cell Permeabilization Kit (Life Technologies, Monza, Italy).

Immuohistochemistry

Formalin-fixed, paraffin-embedded sections were deparaffinized and endogenous peroxidase activity was blocked. Epitope retrieval was performed in 0.01 M citrate buffer, pH 6.0. Mouse monoclonal anti-ENO1 Ab (2 µg/mL, clone 72/1.11, kindly provided by Laboratory of Tumor Immunology, Prof. Francesco Novelli, University of Torino) was diluted 1:1000 and incubated for 1 hour at room temperature. Sections were then incubated with biotinilated anti-mouse secondary Ab and developed with DAB according to the manufacturer's instructions (Dako Spa).

Immunofluorescence microscopy

Formalin-fixed, paraffin-embedded sections were deparaffinized and endogenous peroxidase activity was blocked. Epitope retrieval was performed in 0.01 M citrate buffer, pH 6.0 (40 minutes, 98°C). Ab used were mouse monoclonal anti-ENO1 (2 µg/mL), goat polyclonal anti-CD23 (10 µg/mL, R&D Systems, Minneapolis, MN, USA) and anti-CD3-ε (0.4 µg/mL, Santa Cruz Biotechnology), rabbit polyclonal anti-CD2 (1.3 µg/mL, Sigma Aldrich, Milano, Italy) and anti-Ki67 (5 µg/mL, Abcam, Cambridge, UK). The following secondary Ab were used to visualize the reaction: AlexaFluor-633-conjugated goat anti-mouse IgG (Life Technologies), DyLight-488-conjugated bovine anti-goat IgG and DyLight-594-conjugated donkey anti-rabbit IgG (both from Jackson Immunoresearch, West Grove, PA, USA). Samples were counterstained with 4,6-diamidino-2-phenylindole and mounted in Slow-Fade Gold reagent (both from Life Technologies). Immunofluorescent slides were analyzed using a TCS SP5 laser scanning confocal microscope equipped with 4 lasers (Leica Microsystems); images were acquired with LAS AF Version Lite 2.4 software (Leica Microsystems) and processed with Photoshop (Adobe Systems, San Jose, CA). Staining quantification of ENO1 on CLL cells and T lymphocytes was performed by comparing ENO1 mean pixel intensity on CD23+ vs CD2+ cells. Staining quantification was performed in 63x magnification images of CD23+/Ki67+ vs CD23+/Ki67- cells or CD3+/Ki67+ vs CD3+/Ki67- cells. At least 10 independent measurements of the different areas per slide from 2 different images per sample were analyzed using ImageJ software (National Institutes of Health; http://rsbweb.nih.gov/ij/).

Complement dependent cytotoxicity assay

The anti-CD52 monoclonal Ab alemtuzumab was obtained from the AOU Città della Salute e della Scienza di Torino pharmacy. Complement sources used were sera from our cohort of patients with CLL or HD. Briefly 2 x 10^5 purified CLL cells in RPMI standard medium were pretreated at room temperature for 30 minutes with 10 µg/mL of alemtuzumab or left untreated. These cell suspensions were then split and received either no serum, or 25% and 100% patients' or HD serum. The cells were then incubated for 1 hour at 37°C with 95% humidity and 5% CO_2 and then washed with 2 mL of PBS before analysis. The percentage of viable cells was determined by Annexin-V (AnnV) or AnnV/Propidium Iodide (PI) staining with the MEBCYTO-Apoptosis Kit and by flow cytometry with a FACSCalibur and CELLQuest software (Becton Dickinson). Data were analyzed with FlowJo software (Tree Star, Inc). U937 cells were incubated with patients' serum for 1 hour at 37°C with 95% humidity and 5% CO_2 and then washed with 2 mL of PBS before cell viability analysis.

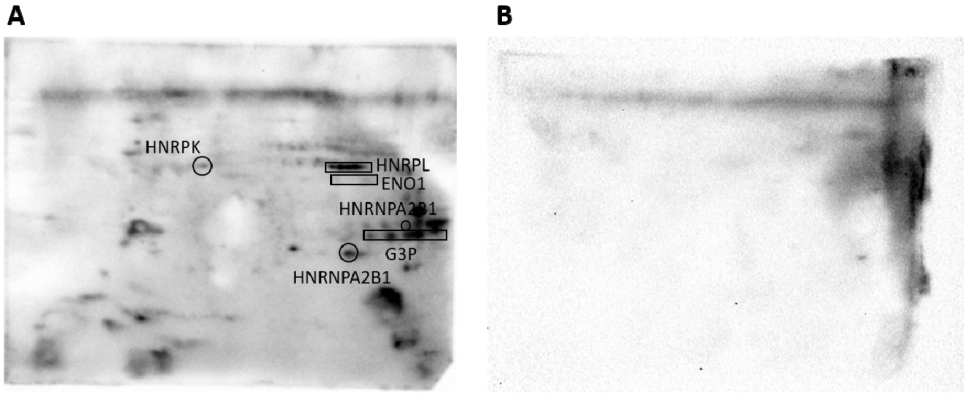

Supplementary Figure S1: Western blots of autologous serum and ScFv-Fc hybridized in parallel on proteomic maps of a CLL patient. Total proteins from purified CLL cells of a representative patient (patient 10) were separated 2-DE, transferred on nitrocellulose membranes for WB and probed with the autologous serum **(A)** or with a soluble derivative of the BCR (ScFv-Fc) **(B).** Patient's serum recognized 5 Ag, while the ScFv-Fc did not produced any spot.

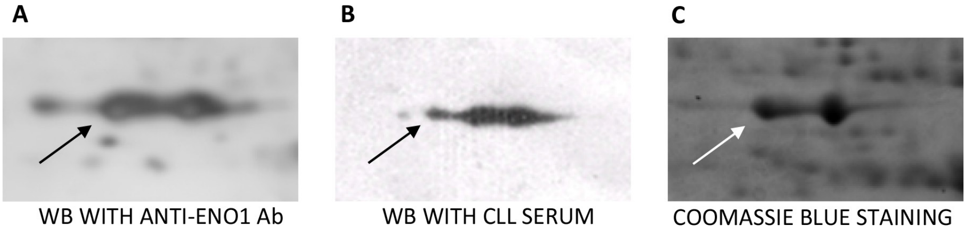

Supplementary Figure S2: Close-up of a 2-DE proteomic map and WB in the location of ENO1 protein. CLL cells lysate proteins were separated by 2-DE, transferred onto nitrocellulose membranes and then immunoblotted with CLL patients serum **(A)** or with an anti-ENO1 rabbit polyclonal Ab **(B).** A Coomassie Blue stained 2-DE proteomic map is also shown **(C).**

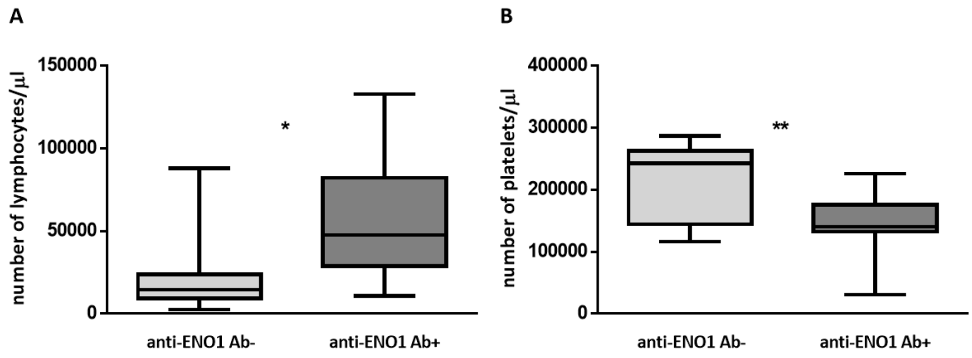

Supplementary Figure S3: Anti-ENO1 Ab reactivity was associated with parameters of progressive disease in patients with CLL. The presence of circulating anti-ENO1 Ab in sera of patients with CLL was significantly correlated with a higher number of lymphocytes **(A)** and a lower number of platelets **(B)** at the time of SERPA (p=0.02 and p=0.007 respectively).

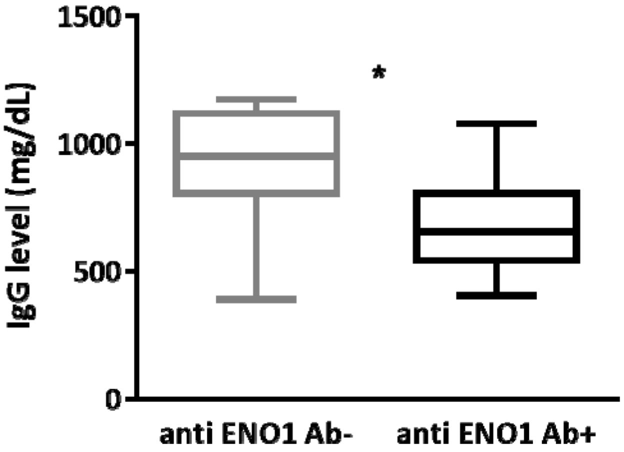

Supplementary Figure S4: Anti-ENO1 Ab reactivity was associated with lower IgG level in patients with CLL. The presence of circulating anti-ENO1 Ab in sera of patients with CLL was significantly correlated with a lower level of serum IgG at the time of SERPA (p=0.02).

Supplementary Table S1: Proteins recognized by (< 3) CLL and HD sera

See Supplementary File 1

Supplementary Table S2: Clinical and biological features of patients with CLL analyzed by SERPA

**No. Patients**	**N = 35[a]**
Median age, years (range)[b]	68 (35-84)
Female, no. (%)	10 (29)
**Binet stage, no. (%)[d]**	
A	20 (65)
B	10 (32)
C	1 (3)
Lymphocytes, no./µl[c, e]	38000 ± 7500
Lymphocytes, %[c, e]	78.5 ± 3.48
Monocytes, no./µl	730 ± 75
Monocytes, %	3,3 ± 0.44
Hb, g/dl[c, g]	13.1 ± 0.4
Plts, no./µl[c, g]	171300 ± 11600
LDH, U/ml[c, h]	410 ± 35
β2M, g/dl[c, i]	1.86 ± 0.16
% B CLL clone[c, j]	77.0 ± 2.7
CD38+, no. (%) [j, k]	10 (31)
ZAP70+, no. (%)[j, l]	6 (19)
K light chain, no. (%)[j]	17 (53)
IGHV mutated, no. (%)[b]	16 (48)
**Cytogenetics by FISH**	
Normal, no. (%)[m]	13 (38)
del(11q) no. (%)[m]	4 (12)
del(13q) no. (%)[m]	8 (24)
del(17p) no. (%)[m]	4 (12)
Progressive disease, no. (%)[b]	10 (30)

Notes:
CLL indicates chronic lymphocytic leukemia; IGHV, immunoglobulin heavy chain variable region; LDH, lactate dehydrogenase; and Plts, platelets.
[a] All cases were evaluable for each of the variables, if not otherwise specified.
[b] Data were available in 33 patients.
[c] Data are expressed as mean ± SEM.
[d] Data were available in 31 patients.
[e] Data were available in 21 patients.
[f] Data were available in 22 patients.
[g] Data were available in 27 patients.
[h] Data were available in 28 patients.
[i] Data were available in 17 patients.
[j] Data were available in 32 patients.
[k] CD38 expression was considered positive when > 30%.
[l] ZAP70 expression was considered positive when > 20%.
[m] Data were available in 34 patients.
