# Supplementary material for: Humoral immune responses toward tumor-derived antigens in previously untreated patients with chronic lymphocytic leukemia
Source: Oncotarget. 2016 Nov 30;8(2):3274–88. doi: 10.18632/oncotarget.13712 (PMC5356881; doi:10.18632/oncotarget.13712)
Supplement: Supplementary file 2 [file oncotarget-08-3274-s002.doc]

| **Table S1. Proteins recognized by (< 3) CLL and HD sera** | | | | | | | | | | |
| --- | --- | --- | --- | --- | --- | --- | --- | --- | --- | --- |
| **MW (kDa)/p***I* | **Swiss-Prot Accession number** | **Protein name** | **Sequence coverage (%)** | **Matched**  **peptides** | **Mascot score** | **Recurrence of antibody (%)** | | | | |
|  |  |  |  |  |  | **CLL patients** | **Healthy donors** | | | **p** |
| 56,525/5.26 | P06576 | ATP synthase subunit beta, mitochondrial (ATPB) | 46 | 18 | 113 | 2 (6) | | 0 | ns | |
| 36,201/5.4 | Q9HC38 | Glyoxalase domain-containing protein 4 (GLOD4) | 46 | 10 | 94 | 2 (6) | | 0 | ns | |
| 40,908/8.73 | Q8IUY3 | GRAM domain-containing protein 2 (GRAM2) | 18 | 6 | 60 | 2 (6) | | 0 | ns | |
| 57,146/5.98 | [P30101](http://www.uniprot.org/uniprot/P30101) | Protein disulfide-isomerase A3 (PDIA3) | 24 | 10 | 109 | 2 (6) | | 0 | ns | |
| 19,834/8.59 | [Q6UWF9](http://www.uniprot.org/uniprot/Q6UWF9) | Protein FAM180A (F180A) | 34 | 5 | 61 | 2 (6) | | 0 | ns | |
| 31,057/5.65 | P60174 | Triosephosphate isomerase (TPIS) | 47 | 12 | 145 | 2 (6) | | 0 | ns | |
| 50,095/4.78 | P07437 | Tubulin β chain (TBB5) | 37 | 20 | 108 | 2 (6) | | 0 | ns | |
| 38,918/6.57 | P04083 | Annexin A1 (ANXA1) | 32 | 9 | 57 | 1 (3) | | 0 | ns | |

| 48,283/4.29 | P27797 | Calreticulin (CALR) | 40 | 14 | 115 | 1 (3) | 0 | ns |
| --- | --- | --- | --- | --- | --- | --- | --- | --- |
| 27,248/5.09 | O00299 | Chloride intracellular channel protein 1 (CLIC1) | 45 | 9 | 110 | 1 (3) | 0 | ns |
| 495,790/6.13 | Q8NCM8 | Cytoplasmic dynein 2 heavy chain 1 (DYHC2) | 6 | 17 | 70 | 1 (3) | 0 | ns |
| 36,197/9.31 | Q93070 | Ecto-ADP-ribosyltransferase 4 (NAR4) | 20 | 5 | 57 | 1 (3) | 0 | ns |
| 53,143/6.77 | P07099 | Epoxide hydrolase 1 (HYEP) | 18 | 7 | 61 | 1 (3) | 0 | ns |
| 67,690/7.18 | Q96AE4 | Far upstream element-binding protein 1 (FUBP1) | 22 | 9 | 88 | 1 (3) | 0 | ns |
| 56,577/8.54 | P02675 | Fibrinogen beta chain (FIBB) | 39 | 17 | 79 | 1 (3) | 0 | ns |
| 38,609/5.39 | Q9UIM3 | FK506 binding protein like (FKBPL) | 16 | 5 | 55 | 1 (3) | 0 | ns |
| 49,484/8.52 | P31943 | Heterogeneous nuclear ribonucleoprotein H (HNRNPH1) | 23 | 5 | 60 | 1 (3) | 0 | ns |
| 20,992/5.12 | Q04760 | Lactoylglutathione lyase (LGUL) | 42 | 6 | 73 | 1 (3) | 0 | ns |
| 38,760/5.82 | P40121 | Macrophage-capping protein (CAPG) | 29 | 7 | 64 | 1 (3) | 0 | ns |
| 44,985/8.3 | P00558 | Phosphoglycerate kinase 1 (PGK1) | 40 | 11 | 109 | 1 (3) | 0 | ns |
| 15,216/8.44 | P07737 | Profilin-1 (PROF1) | 39 | 6 | 66 | 1 (3) | 0 | ns |
| 20,050/6.33 | Q99497 | Protein DJ-1 (PARK7) | 68 | 10 | 120 | 1 (3) | 0 | ns |
| 554,704/6.09 | Q9Y6U9 | Protein piccolo (PCLO) | 4 | 16 | 64 | 1 (3) | 0 | ns |
| 13,291/5.71 | P06702 | Protein S100-A9 (S10A9) | 74 | 8 | 88 | 1 (3) | 0 | ns |
| 42,936/5.18 | P35237 | Serpin B6 (SPB6) | 25 | 7 | 58 | 1 (3) | 0 | ns |
| 48,283/5.63 | Q8NBS9 | Thioredoxin domain-containing protein 5 (TXND5) | 23 | 7 | 67 | 1 (3) | 0 | ns |
| 30,868/8.62 | P21796 | Voltage-depending anion-selective channel protein 1 (VDAC1) | 38 | 6 | 80 | 1 (3) | 0 | ns |
| 137,343/7.10 | Q2M389 | WASH complex subunit 7 (WASH7) | 11 | 8 | 60 | 1 (3) | 0 | ns |
| 179,777/8.67 | Q5JPB2 | Zinc finger protein 831 (ZN831) | 10 | 9 | 60 | 1 (3) | 0 | ns |

MW, Molecular weight; p*I*, isoelectrical point. Sequence coverage (%) is the percentage of the protein primary sequence covered by peptides obtained by tripsyn digestion of the Ag spot. Matched peptides is the rate between the number of peptides corresponding to the primary sequence of the protein and the total number of peptides analyzed. Mascot score is a measure of the corrispondence of peptides to the protein primary sequence.
